# Genome Sequence Analysis of *In Vitro* and *In Vivo* Phenotypes of Bunyamwera and Ngari Virus Isolates from Northern Kenya

**DOI:** 10.1371/journal.pone.0105446

**Published:** 2014-08-25

**Authors:** Collins Odhiambo, Marietjie Venter, Konongoi Limbaso, Robert Swanepoel, Rosemary Sang

**Affiliations:** 1 Human Health Division, International Centre of Insect Physiology and Ecology, Nairobi, Kenya; 2 Zoonoses Research Unit, Department Medical Virology, University of Pretoria, Pretoria, South Africa; 3 Centre for Virus Research, Kenya Medical Research Institute, Nairobi, Kenya; 4 Division of Emerging Infectious Disease, United States Army Medical Research Unit, Nairobi, Kenya; University of Texas Medical Branch, United States of America

## Abstract

Biological phenotypes of tri-segmented arboviruses display characteristics that map to mutation/s in the S, M or L segments of the genome. Plaque variants have been characterized for other viruses displaying varied phenotypes including attenuation in growth and/or pathogenesis. In order to characterize variants of Bunyamwera and Ngari viruses, we isolated individual plaque size variants; small plaque (SP) and large plaque (LP) and determined *in vitro* growth properties and *in vivo* pathogenesis in suckling mice. We performed gene sequencing to identify mutations that may be responsible for the observed phenotype. The LP generally replicated faster than the SP and the difference in growth rate was more pronounced in Bunyamwera virus isolates. Ngari virus isolates were more conserved with few point mutations compared to Bunyamwera virus isolates which displayed mutations in all three genome segments but majority were silent mutations. Contrary to expectation, the SP of Bunyamwera virus killed suckling mice significantly earlier than the LP. The LP attenuation may probably be due to a non-synonymous substitution (T858I) that mapped within the active site of the L protein. In this study, we identify natural mutations whose exact role in growth and pathogenesis need to be determined through site directed mutagenesis studies.

## Introduction

Bunyamwera virus is the prototype virus of the *Orthobunyavirus* genus of the *Bunyaviridae* family of arboviruses. The virus is associated with febrile illness with headache, arthralgia, rash and infrequent central nervous system involvement [1]. While viruses of the *Orthobunyavirus* genus are known to cause human disease, they were previously not associated with hemorrhagic manifestations. However, Ngari virus has been implicated in recent outbreaks of hemorrhagic fevers in Kenya and Somalia [2]–[4]. Ngari virus is thought to have arisen through genetic reassortment between two bunyaviruses co-circulating within the same environment [4].

Like other viruses within the *Bunyaviridae* family, the Bunyamwera virus genome consists of three negative-sense RNA segments that employ a variety of coding strategies leading to generation of a limited set of structural and non-structural proteins [5], [6]. The L (large) segment encodes a large protein that comprises the RNA-dependent RNA polymerase, for replication and transcription of genomic RNA segments. The M (medium) segment encodes a precursor polypeptide which yields the viral surface glycoproteins Gn and Gc, and a nonstructural protein (NSm), and the S (small) segment encodes the nucleocapsid (NC) and a nonstructural protein (NSs) in overlapping reading frames [6]. The prevalence of members of the *Bunyaviridae* family are likely underestimated because of the lack of detection tools arising partly from their high level of diversity, limited phenotypic and genetic characterization and segmented nature of their genome.

Orthobunyaviruses are mostly isolated and amplified in interferon defective African green monkey kidney epithelial Vero cell line that may result in mutations yielding substrains that are phenotypically different from the parental wild type virus [7]. Such observations have been reported among other viruses of the family *Bunyaviridae*, including Puumala virus in which the large plaque (LP) grows to higher titers than the small plaque (SP) and the parental wild type (WT) virus [7]. Genome sequencing analysis revealed differences at two positions in the NC protein and two positions in the L protein [7]. Attenuation, both *in vivo* and *in vitro* has also been observed for the SP of West Nile virus [8]. Attenuated pathogenesis of substrains of Dengue and Japanese encephalitis virus has also been reported in mice experiments [9]–[12]. Thus, understanding the genetic diversity in a heterogeneous arbovirus population is important, given that any variant can be favored by selection which ultimately affects fitness. We hypothesize that natural mutations may accumulate during passage of Bunyamwera and Ngari viruses, obtained from entomological surveillance in Kenya [13]. Such mutations may yield substrains with genotypic and phenotypic differences between each other and with the parental WT strains. In analyzing the viral phenotypes, we determined the kinetics of replication following infection of Vero cells. Additionally, we demonstrated pathogenesis of viral strains after intraperitoneal inoculation of mice. We report that Bunyamwera and Ngari virus substrains display contrasting phenotypes compared with each other and to the parental wild type.

## Methods

### Ethics statement

The study protocol (number SSC 2677) was approved by the Animal Care and Use Committee of the Kenya Medical Research Institute and by the Animal Ethics Committee of the University of Pretoria (Protocol number H012-13). All animal experiments were carried out in accordance with the regulations and guidelines of the Kenya Medical Research Institute and University of Pretoria Animal Ethics Committees.

### Virus stock preparation

The sites in Kenya and vector species from which the 5 virus isolates used in the study were obtained is summarized in Table 1. Vero cells (CCL-81, ATCC) were grown in T-75 culture flasks containing Eagle's minimum essential medium (Sigma) (MEM) supplemented with 10% fetal bovine serum (Gibco-BRL), 2% L-glutamate (Sigma) and 2% penicillin/streptomycin (Gibco-BRL). Confluent cells were rinsed with sterile phosphate buffered saline (PBS), and 0.1 mL clarified homogenate of field collected mosquitoes were added followed by incubation at 37°C for one hour with constant rocking to allow virus adsorption. After incubation, maintenance medium (MEM with Earle's salts, 2% FBS, 2% glutamine, 100 U/mL penicillin, 100 µg/mL streptomycin, and 1 µL/mL amphotericin B) was added, cells incubated at 37°C and observed daily for cytopathic effects (CPE). Each isolate was grown individually to avoid cross-contamination and supernatants were harvested when approximately 75% of the cells exhibited CPE. The culture supernatants were aliquoted and stored at −80°C until used. The stock concentrations were determined by plaque assay titration.

**Table 1:** Virus isolates obtained from diverse geographical regions and species in Kenya.

Specimen identity	Isolate code	Isolation site	Isolation date	Mosquito/tick species	Passage history
**Bunyamwera viruses**	GSA/S4/11232	Garissa	2009	*Aedes mcintoshi*	Vero 3
	MGD/S1/12060	Magadi	2010	*Anopheles funestus*	Vero 3
**Ngari viruses**	TND/S1/19801	Tana-delta	2011	*Anopheles funestus*	Vero 3
	GSA/TS7/5170	Garissa	2009	*Amblyomma gemma*	Vero 3
	ISL/TS2/5242	Isiolo	2009	*Rhipicephalus pulchellus*	Vero 3

### Plaque assay and purification

Vero cells were seeded on 6 well plates and incubated in a humidified CO_2_ incubator at 37°C overnight before use. The cells were used when they attained 75–90% confluence. Ten-fold dilutions of the virus isolates were prepared in maintenance media. Media was carefully aspirated from the wells using sterile transfer pipettes and 100 µl of the appropriate viral dilution added to each of duplicate wells of 6-well plates with gentle rocking to evenly distribute the virus. Plates were incubated at 37°C for 1 hour after which media was carefully aspirated and 3 ml of 1.25% methylcellulose solution gently added to each plate. Plates were placed in a CO_2_ humidified incubator and incubated for 5 days. Development of plaques was monitored by visualization under an inverted microscope. To facilitate visualization of plaques, methylcellulose solution was carefully aspirated using transfer pipette followed by fixation in 10% formaldehyde after which plates were stained with crystal violet solution.

Bunyamwera and Ngari virus isolates (previously passaged 3 times on Vero cells) with a titer of 1×10^9^ PFU/ml were diluted in maintenance media to approximately 10 PFU/ml. Confluent Vero cells in 24-well plates were infected with 100 µl of diluted virus per well. After adsorption for 1 h at 37°C, the cells were overlaid with 1.25% methylcellulose. Five days later, the methylcellulose medium was carefully aspirated and sterile pasture pipettes used to pick plaques from wells with single plaques and placed in 500 µl of maintenance media. The plaque phenotypes were then propagated on Vero cells and the procedure repeated twice more, without intermediate amplification, for each of the plaque isolates. The purified isolates were then amplified by propagation on confluent Vero cells in flasks and then frozen at −80°C until use.

### *Invitro* growth kinetics

The viral isolates including the parental WT, i.e. mixture of SP and LP, were used to infect 90% confluent monolayers of Vero cells at a multiplicity of infection of 0.01 and incubated for one hour to allow virus adsorption. Infected monolayers were washed twice with sterile PBS and overlaid with maintenance medium and incubated at 37°C. An aliquot of tissue culture fluid (0.5 ml) was collected every 12 hours for the first 2 days and once on day 3 of infection, mixed 1∶10 with maintenance media and frozen at −80°C until use. Daily samples were titrated by plaque assay as described above. The statistical package R (R Development Core Team, 2008) [14] was used for fitting exponential growth data using the Kruskal–Wallis test. The detection of correlated error structure in the growth curve data was carried out as follows; the log-transformed data was fit to linear mixed effects models using R, and an AR1 model was determined to fit the data better than a repeated measures model.

### Molecular characterization of plaque purified phenotypes

#### Virus isolation and cDNA synthesis

For RNA extraction, the MagNA Pure LC RNA Isolation Kit I (Roche Diagnostics) was used. Complementary DNA (cDNA) was synthesized using Transcriptor First Strand cDNA Synthesis Kit (Roche Applied Science) with Random hexamers followed by PCR using Phusion High-Fidelity PCR Kit (Finnzyme OY, Espoo, Finland) and appropriate primers. Primers for each segment were either designed based on sequences of Bunyamwera, Batai and Ngari viruses available in GenBank or obtained from previous publications [15], [16] (table S1). Amplified DNA fragments were visualized by electrophoresis on a 1.5% agarose gel. The Amplified DNA was purified and prepared for sequencing using ExoSAP-IT PCR clean-up kit (USB Corp, Cleveland, OH) according to the manufacturer's instructions and stored at −20°C.

#### Sequence analysis of viral genomes

Sequencing was performed using different sets of primers for the S, M and L segments as defined above using Big Dye V3.1 kit (Applied biosystems) and injection on a 3500XL genetic analyser (Foster city, California, USA). The sequences obtained were cleaned and edited using Bioedit software (www.mbio.ncsu.edu/BioEdit/BioEdit.html), USA for both the reads from the forward and reverse primers. Sequences obtained were compared to those in the gene bank using the Basic Local Alignment Search tool (BLAST) [17] in NCBI GenBank (http://www.ncbi.nlm.nih.gov/blast/Blast) to identify similar sequences. The clean sequences of each segment of each phenotype were aligned against the corresponding segment sequences of the wild type virus isolate using Bioedit. Nucleotide and amino acid similarity and diversity between the virus phenotypes were computed in MEGA v5.20 [18] using the p-distance method.

### Clinical disease in mice

Pathogenicity of the plaque phenotypes was evaluated in Swiss Albino suckling mice (1–4 days old) and 6 week old mice. Mice were inoculated intraperitoneally with 100 µl of 10^9^ PFU/ml of selected wild type or amplified plaque purified virus substrains in maintenance media. All mice were carefully observed twice daily, up to 14 days for clinical disease which included characteristic tremors and hind-limb paralysis. Survival functions were graphed for the two sets of viruses. Pairwise comparisons of survival curves were made using the Wilcoxon-Breslow test to test for equality of survivor functions.

## Results

### Isolation and Purification of Plaque phenotypes

**Figure 1:**
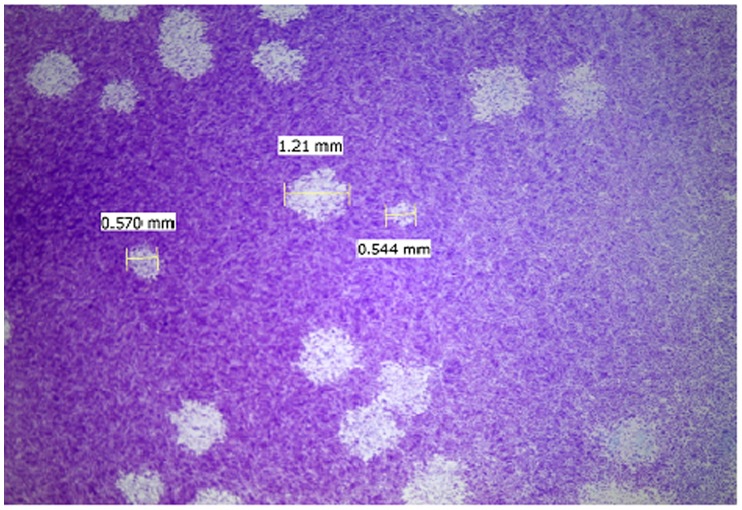
Photograph of virus infected Vero cell monolayer showing plaque phenotypes based on plaque size. Vero cell monolayers were fixed in 0.5% crystal violet solution. Plaque size was measured using a Zeiss microscope.

Plaque titration of Ngari and Bunyamwera viruses yielded plaques of two significantly distinct phenotypes, large plaques (LP) (Range: 0.88–1.21 mm) and small plaques (SP) (Range: 0.47–0.66 mm) (Figure 1). Each plaque phenotype was sub-cloned twice and purified each time by inoculation onto new Vero cells. The plaque phenotypes retained their plaque size after amplification by single passage in cell culture to generate viral stocks with high titers for onward experimentation. The cloned LP substrain produced larger plaques than the SP suggesting that the former was more efficiently replicated in Vero cells than the latter.

### *In vitro* growth curves

**Figure 2:**
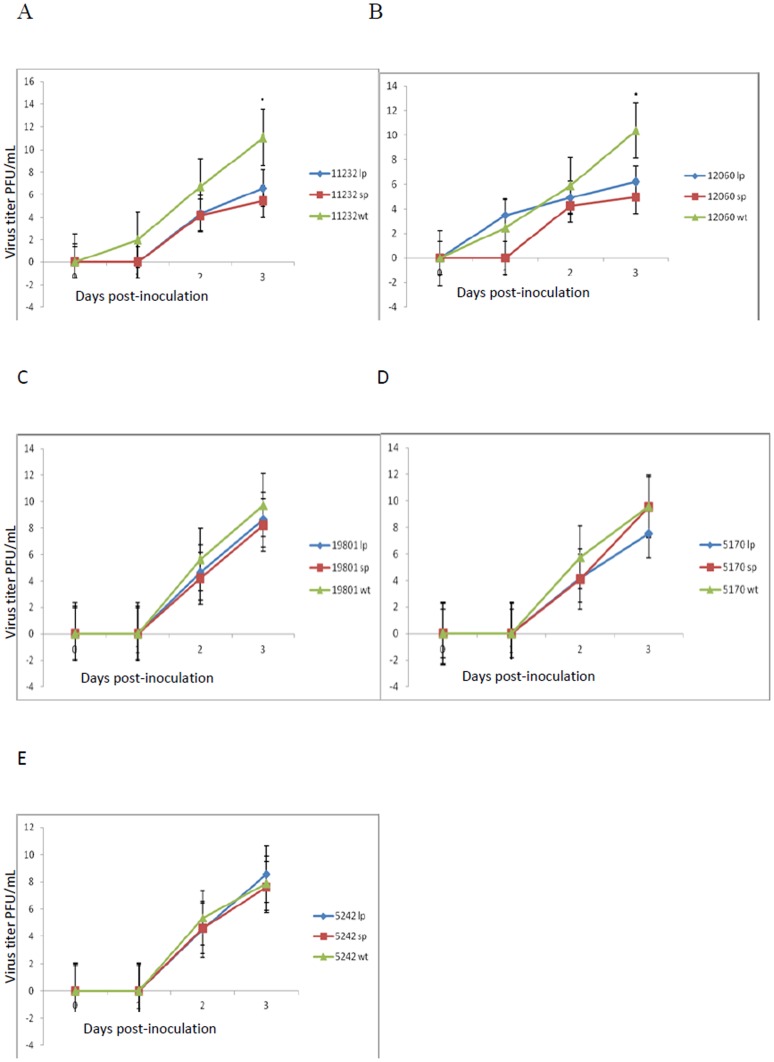
Growth kinetics of wild type parental and amplified Plaque purified phenotypes of A–B) Bunyamwera and C–E) Ngari virus isolates. The viral isolates including the parental WT, i.e. mixture of SP and LP, were used to infect 90% confluent monolayers of Vero cells at a multiplicity of infection of 0.01. Aliquots of tissue culture fluid were collected at different timepoints and titers determined by plaque assay. The experiment was replicated thrice. The statistical package R was used for fitting exponential growth data using the Kruskal–Wallis test. The detection of correlated error structure in the growth curve data was carried out as follows; the log-transformed data was fit to linear mixed effects models using R, and an AR1 model was determined to fit the data better than a repeated measures model.

In general, the LP phenotype of both Bunyamwera virus isolates grew at a faster rate and to a significantly higher titer (p = 0.009) than the SP phenotype by day 3 of infection (Figure 2A and 2B). The Bunyamwera WT reached approximately 5-logs higher than the virus titer of the SP and LP phenotypes by day 3 post-infection. However, the difference in growth of the SP and LP phenotypes was insignificant. Bunyamwera virus WT isolates generally grew to a higher titer than Ngari virus WT isolates.

For Ngari virus isolates (Figures 2C–E), the difference in titer between the WT and plaque phenotypes at 3 days post-infection was not more than 1 log except for isolate GSA/S7/5170 (Figure 2D). However, the difference in titer between the WT and plaque variants was not significant.

### Genetic characterization of plaque phenotypes

Comparison of the nucleotide and amino acid sequences of low passage Ngari virus isolates revealed little or no divergence within the S segments (Table 2). The N and NSs proteins of Ngari virus isolates were 100% conserved between the phenotypes. However, all Bunyamwera virus isolate phenotypes exhibited nucleotide substitutions in all segments compared to the WT except the L segment of isolate GSA/S4/11232SP. There was a single nucleotide change in the M segment of isolate GSA/TS7/5170SP and ISL/TS2/5242LP and three changes on the L segment of isolate ISL/TS2/5242SP. All these single nucleotide changes were synonymous. There were no changes in nucleotide sequences of L segments of other Ngari virus isolates except isolate TND/S1/19801LP which had one nucleotide substitution resulting in a non-synonymous change in the amino acid sequence (D84N).

**Table 2:** Nucleotide differences between wild type parental and Plaque purified phenotypes of Bunyamwera and Ngari virus isolates.

Virus identity	Virus Phenotype	Passage history	Nucleotide (amino acid) substitution in indicated virus segment		
			S	M	L
**Bunyamwera viruses**	**GSA/S4/11232 SP**	Vero 7	G320C (G79R)	A1503G (K485E)	No changes
				C2009T	
	**GSA/S4/11232 LP**	Vero 7	T244C	G3593A	G129A (A27T
			C559T		T1523C
					C2623T (T858I)
	**MGD/S1/12060 SP**	Vero 7	A803T	C82T (A11V)	G278T
			C915T	G1503A (E485K)	A6099C (Q217K)
				T2601C (S853P)	
				G3423A (E1127K)	
				T4229C	
	**MGD/S1/12060 LP**	Vero 7	A803T	C1319T	A6099C(Q217K)
			C915T	G1503A (E485K)	
				T4229C	
**Ngari viruses**	**TND/S1/19801 SP**	Vero 7	No changes	No changes	No changes
	**TND/S1/19801 LP**		No changes	No changes	G300A (D84N)
	**GSA/TS7/5170 SP**		No changes	T3272C	No changes
	**GSA/TS7/5170 LP**		No changes	No changes	No changes
	**ISL/TS2/5242 SP**		No changes	No changes	G869A
					G2741A
					G2993A
	**ISL/TS2/5242 LP**		No changes	G3839A	No changes

* LP = large plaque; SP = small plaque.

For the Bunyamwera virus isolates, there were more transversions than transitions resulting in several non-synonymous codons.

### Mice pathogenesis

**Figure 3:**
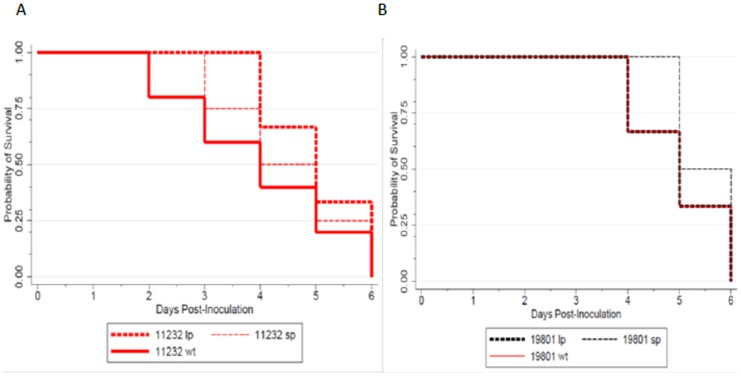
Survival curves of mice following infection with wild type parental and amplified plaque purified phenotypes of A)Bunyamwera (GSA/S4/11232) and B)Ngari (TND/S1/19801) virus isolates. Groups of mice (n = 12) were inoculated with 10^9^ PFU/ml of virus and observed for signs of clinical illness. The experiment was replicated three times. Survival functions were graphed for the two sets of viruses. Pairwise comparisons of survival curves were made using the Wilcoxon-Breslow test to test for equality of survivor functions.

We selected the WT and plaque phenotypes of isolates GSA/S2/11232 and TND/S7/19801 for the mice pathogenesis experiments. Six-week old mice were not susceptible to infection by either virus isolate. However, newborn mice were susceptible to infection with both virus isolates and displayed clinical symptoms such as hind limb paralysis, tremors, disorientation and mortality beginning 2–3 days post inoculation. By day 4 post-infection, mice inoculated with isolate GSA/S4/11232SP had a 50% probability of survival compared to the LP phenotype that had approximately 70% survival probability (Figure 3A). The difference in survival probability between the SP and LP phenotypes was significant (p = 0.011). The converse was true for mice inoculated with Ngari virus isolate TND/S1/19801 where the LP phenotype was more lethal than the SP phenotype. Mice inoculated with the LP phenotype had a survival probability below 75% by day 4 and below 50% by day 5 post-infection whereas the SP phenotype had a 100% and 50% survival probability at day 4 and 5 post-infection respectively (Figure 3B). However, the difference in mortality was not significant (p = 0.3579).

## Discussion

In the current study we evaluate the genetic diversity of plaque purified phenotypes of Bunyamwera and Ngari virus isolates by gene sequencing. We also determine the rate of *in vivo* growth in Vero cells and evaluate pathogenesis of the viral phenotypes in Swiss Albino mice. Difference in growth was more pronounced in Bunyamwera than Ngari virus isolates. This may be explained by the more mutations observed for the former possibly due to the extra passages which the SP and LP phenotypes underwent compared to the WT. In contrast, Ngari virus phenotypes had fewer mutations despite undergoing extra passages than the WT during the purification and amplification processes. As expected, the LP phenotypes grew to a higher titer than the SP phenotypes for both Bunyamwera and Ngari virus isolates. Previous studies of other viruses have correlated plaque size to replication rate with the LP phenotypes displaying faster replication rate than the SP phenotype [19]–[21]. The LP phenotypes were generally more virulent than SP phenotypes and would be expected to be the same both *in vitro* and *in vivo* on the assumption that LP phenotypes produce larger foci of cell destruction [22]. However, inoculation of mice with selected Bunyamwera and Ngari virus isolate phenotypes resulted in discordant observations in the present study. While mice inoculated with the SP phenotype of Ngari virus isolate TND/SA/19801 survived longer than the LP phenotype, the reverse was true for Bunyamwera virus isolate GSA/S4/11232 in which mice inoculated with the SP phenotype died 3 days post-inoculation compared to 4 days post-inoculation for the LP phenotype and this difference in mortality rate was significant. This difference in neurovirulence for phenotypes of Bunyamwera virus isolate GSA/S4/11232 in mice cannot fully be accounted for by the rate of replication as shown in the one-step growth curves. Previous neurovirulence studies of viruses within the *Orthobunyavirus* genus have mapped such differences to the L segment [23]. The study by Endres et al., was designed to identify molecular determinants responsible for attenuation of a variant California serogroup virus. Another study investigating the biological function of Bunyamwera L protein demonstrated that mutations in the polymerase genome affect the ability of Bunyamwera virus to replicate in different cells [24]. Thus, the discordance observed in the current study may have been dependent on the single nucleotide substitutions that were present in the different segments of the Bunyamwera virus isolate.

It is interesting that all the nucleotide substitutions on the M segment while resulting in non-synonymous amino acid changes, involved substitution of amino acids with similar properties, thus, a significant difference in protein function would be unexpected. The M segment substitutions resulted in exchange of positively charged amino acids, glutamic acid for lysine. However, two mutations in the L segment of the LP phenotype involved substitution of amino acids with different properties which are likely to alter the functionality of the L protein. Thus for isolate GSA/S4/11232, the LP attenuated pathogenesis may be mapped to any of the 2 non-synonymous mutations on the L segment. The T858I mutation resulting in amino acid substitution of a polar for a non-polar amino acid, occurring within the predicted catalytic site of the L protein (AA 597-1330) seems the most plausible cause of the observed attenuation in pathogenesis. Mutation within the catalytic site of the L protein has been demonstrated to abolish polymerase activity in a previous study [24].

With regard to isolate TND/S1/19801, the SP phenotype, which was attenuated in mice but genetically similar to the WT virus, it is likely that this phenotype was present in a higher quantity in the WT virus, which is a mixture of both LP and SP phenotypes, and could have preferentially been sequenced. However, in the mice pathogenesis experiment, it is likely that the LP phenotype in the WT grew at a faster rate as expected and resulted in earlier death of mice compared to the SP phenotype. However, we did not isolate the infecting virus from mice to confirm this observation. Another limitation was the use of interferon defective Vero cells for the one step growth curve analysis which may have limited our comparison with mice pathogenesis as the GSA/S4/11232 SP phenotype may have been better at counteracting the interferon response. Additionally, we did not sequence the non-coding regions of the genomic segments which have been documented to play a role in virus growth and pathogenesis [25]–[27].

In summary, we have identified a mutation in the L segment of Bunyamwera virus isolate GSA/S4/11232 LP phenotype which may be associated with decreased pathogenesis in suckling mice and virus replication in Vero cells. In addition, we have identified other natural mutations whose role in viral growth and pathogenesis should be determined. Site directed mutagenesis studies may clarify the exact mutation involved in the observed phenotypic changes.

## Supporting Information

Table S1 **Primers used in sequencing of Kenyan Bunyamwera and Ngari virus isolates.** Primers for each segment were either designed based on conserved regions of sequences of Bunyamwera, Batai and Ngari viruses available in GenBank or obtained from previous publications.
(DOCX)
