# Supplementary material for: Genome Sequence Analysis of In Vitro and In Vivo Phenotypes of Bunyamwera and Ngari Virus Isolates from Northern Kenya
Source: PLoS One. 2014 Aug 25;9(8):e105446. doi: 10.1371/journal.pone.0105446 (PMC4143288; doi:10.1371/journal.pone.0105446)
Supplement: Table S1 — Primers used in sequencing of Kenyan Bunyamwera and Ngari virus isolates. Primers for each segment were either designed based on conserved regions of sequences of Bunyamwera, Batai and Ngari viruses available in GenBank or obtained from previous publications. (DOCX) [file pone.0105446.s001.docx]

Table S1: Primers used in sequencing of Kenyan Bunyamwera and Ngari virus isolates. Primers for each segment were either designed based on conserved regions of sequences of Bunyamwera, Batai and Ngari viruses available in GenBank or obtained from previous publications.

| **Target virus** | **Target gene/protein** | **Primer sequence pair (5’-3’)** | **Position** | **Reference** |
| --- | --- | --- | --- | --- |
| **Bunyamwera** | **S segment** | BUNS1 (AGTAGTGTACTCCACACTACAAACT)  and BUNS9 (AGGAATCCACTGAGGCGGTGGAGG) | 1-25 to 358-381 | Yandoko et al, 2007 |
|  |  | BUNS4 (CTGGCAACCGGAACAACCCAGTT)  and BUNS5 (GAGACAACTGTCAGTGCAGACTGAA) | 318-340 to 687-711 | Yandoko et al, 2007 |
|  |  | BUNS10 (TCAGTCTGCACTGACAGTTGTCTC)  and BUNS2 (AGTAGTGTGCTCCACCTAAAACTTA) | 688-711 to 937-961 | Yandoko et al, 2007 |
|  | **Polyprotein M segment** | Bunya M14C (CGGAATTCAGTAGTGTACTACC) and Bunya M619 (GACATATGYTGATTGAAGCAAGCATG) | 1-14 to 586-576 | Yanase et al, 2006) |
|  |  | BUNM11F (TCAGCACTGGCTGGTTTAAG)  and BUNM11R (ACCTGCACCGAAGAGTGATG) | 481-500 to 1159-1178 | This study |
|  |  | BunM12F (GAGATAGCAGATGTCCTTCAAGC)  and BunM12R (CAAGCAAGTGCATTCTGTGG) | 1092-1114 to 1706-1725 | This study |
|  |  | Bun M3F (TTCACTAATAAATGTGGGTTTTG)  and Bun M3R (ATGCTGACTGCCTGATAGGG) | 1311-1333 to 2052-2071 | This study |
|  |  | Bun M4F (TCCAGGCACTGCTTATGTTC)  and Bun M4R (TCTTCCCCTGGTATGTGGTC) | 2003-2022 to 2652-2671 | This study |
|  |  | Bun M5F (TGTGATTCTGGTCCGCTCTAC)  and Bun M5R (GCATCCCCATCTGCTAGTTC) | 2625-2645 to 3217-3236 | This study |
|  |  | Bun M6F (CAAAGCATGACGAACATTGC)  and Bun M6R (TCAAGGCTGCAGATTGTGTC) | 3136-3155 to 3900-3919 | This study |
|  |  | Bunc7MF (CCAGATTGATGCGAAATGTG)  and BAT 3’ end R (GAATTCAGTAGTGTGCTACC) | 3821-3840 to 4445-4458 | This study |
|  | **L Protein** | M13 BunL 1C (TGTAAAACGACGGCCAGTAGTGTACTCCT)  and BunL605R (RGTGAARTCNCCATGTGC) | 1-14 to 614-597 | Yanase et al, 2006) |
|  |  | Bun2LF (GTTGCTGGACAAGTTTGCTG)  and Bun2LR (TTGCCAATCTTAACCGCTTC) | 551-570 to 1200-1219 | This study |
|  |  | Bun3LF (AAAATGATTGCCAGGTCAAC)  and Bun3LR (TATGAAAAGCCCAGGTGATG) | 1152-1171 to 1870 -1889 | This study |
|  |  | Bun5LF (AAGAAACAACATGTCAATCTACC)  and Bun5LR (TAGCGGATTTCCTCTTCTGC) | 2421-2443 to 3066-3085 | This study |
|  |  | Bunya L For (CAATATAATAGACATAATACATTTAGAGT)  and Bunya L Rev (CTCCATTTDGACATRTCTGCA) | 1617-1645 to 3175-3154 | This study |
|  |  | BUNL6F (GCTGAACACGGACGAGATG)  and BUNL6R (GGCCCAATGTAAGCACAATC) | 3003-3020 to 3765-3784 | This study |
|  |  | BUNL7F (GGAGAACCACTATCTGTCTTTGG)  and BUNL7R (ATTGTGCTGGGTTTTGGATG) | 3717-3739 to 4472-4491 | This study |
|  |  | BUNL8F (GCAGTTCATGCAATCTGTCC)  and BUNL8R (TGTTGCCCTGAATCAATGTG) | 4412-4431 to 5144-5163 | This study |
|  |  | BUNL9F (TCACAGCTGCAAACACTTCC)  and BUNL9R (TCAATCTGCTTGCCTCTTCC) | 4729-4748 to 5315-5334 | This study |
|  |  | BUNL10F (AAGCGAGCAAATAATAGCAAATG)  and BUNL10R (TACCTAGGGCCTCTGGATTG) | 5256-5274 to 5866-5847 | This study |
|  |  | BUNL11F (CCGGCTATTTAAGATCAATAAGG)  and BUNL11R (TGCTCATCGCCATTACATTC) | 5575-5597 to 6168-6187 | This study |
|  |  | BUNL12F (TCTAGCATTGTCCCGTTTTG)  and BUNL12R (TTTTGCCACATAGTGCTTTTG) | 6138-6157 to 6803-6823 | This study |
|  |  | BUNL12F (TCTAGCATTGTCCCGTTTTG)  and Bun 3’end LR (GTAAAACGACGGCCAGTAGTGTGCTCC) | 6138–6157 to 6863–6875 | This study |
| **Ngari virus** | **Polyprotein M segment** | BunyaM14C (CGGAATTCAGTAGTGTACTACC)  and TrialR2 (TGACCCGCAATTTGTAAAGG) | 1-14 to 826-845 | This study |
|  |  | BATM13F (CCAAACCGAGAAGTTGAACC)  and BATM13R (AATCCTTCCAGGACATCAGC) | 419-438 to 1080-1099 | This study |
|  |  | BATM14F (TGCTCATGCTGTGGTCTAGC)  and BATM14R (ACCTCCACTTTGCCTGTGAG) | 798-817 to 1769-1788 | This study |
|  |  | BATAIM3F (CCTGGGGAAGCATTGTGATTACT)  and BATAIM3R (CTAGCCAGCGACTCTTGCCTTCC) | 1704–1726 to 2206–2228 | Jost et al, 2011 |
|  |  | BATAIM4F (GTCGCTGGCTAGTGCTACCTCTGG)  and BATAIM4R (CTGATTATTGTCGGATTTATTGGGAACCT) | 2217–2240 to 2698–2726 | Jost et al, 2011 |
|  |  | BATAIM5F (AAAGGTTCCCAATAAATCCGACAA)  and BATAIM5R (CAAATTCTTCACATCCCCAACGACTA) | 2696–2719 to 3195–3220 | Jost et al, 2011 |
|  |  | BATAIM6F (AGAATTTGGGTGCCTTGCTGTCA)  and BATAIM6R (AGATGTTTGGTCCCCTGTGCTTATTT) | 3213–3235 to 4061–4086 | Jost et al, 2011 |
|  |  | BAT 3’end F (TGTTCGCAGATAACCATGAAAC)  and BAT 3’end R (GAATTCAGTAGTGTGCTACC) | 3688-3709 to 4425-4438 | This study |
|  | **L segment** | NgariL6F (TTTTGAGAAGGAATAATGAAGG)  and NgariL6R (CCCCTGCTGCAACTAACTTC) | 5821-5842 to 6320-6339 | This study |
